# Methylenetetrahydrofolate reductase tagging polymorphisms are associated with risk of esophagogastric junction adenocarcinoma: a case-control study involving 2,740 Chinese Han subjects

**DOI:** 10.18632/oncotarget.22845

**Published:** 2017-12-01

**Authors:** Guowen Ding, Yafeng Wang, Yu Chen, Jun Yin, Chao Liu, Yu Fan, Hao Qiu, Weifeng Tang, Shuchen Chen

**Affiliations:** ^1^ Department of Cardiothoracic Surgery, Affiliated People’s Hospital of Jiangsu University, Zhenjiang, Jiangsu Province, China; ^2^ Department of Cardiology, The People’s Hospital of Xishuangbanna Dai Autonomous Prefecture, Jinghong, Yunnan Province, China; ^3^ Department of Medical Oncology, Fujian Cancer Hospital, Fujian Medical University Cancer Hospital, Fuzhou, Fujian Province, China; ^4^ Department of Medical Oncology, Affiliated People’s Hospital of Jiangsu University, Zhenjiang, Jiangsu Province, China; ^5^ Department of Immunology, School of Medicine, Jiangsu University, Zhenjiang, Jiangsu Province, China; ^6^ Department of Thoracic Surgery, Fujian Medical University Union Hospital, Fuzhou, Fujian Province, China

**Keywords:** MTHFR, polymorphism, esophagogastric junction adenocarcinoma, risk

## Abstract

In this study, we aimed to determine the potential association of *MTHFR* tagging single nucleotide polymorphisms (SNPs) with risk of developing esophagogastric junction adenocarcinoma (EGJA). *MTHFR* rs1801133 G>A, rs3753584 T>C, rs4845882 G>A, rs4846048 A>G and rs9651118 T>C polymorphisms were genotyped in 1,677 healthy individuals and 1,063 patients with EGJA. We found that *MTHFR* rs1801133 G>A polymorphism was significantly associated with the risk of developing EGJA (AA *vs.* GG: adjusted *P* = 0.001; GA/AA *vs.* GG: adjusted *P* = 0.007 and AA *vs.* GA/GG: adjusted *P* = 0.001). However, for *MTHFR* rs4845882 G>A polymorphism, the decreased risk of EGJA was found in two genetic models (AA *vs.* GG: adjusted *P* = 0.002 and AA *vs.* GA/GG: adjusted *P* = 0.005). In addition, for *MTHFR* rs3753584 T>C and rs9651118 T>C polymorphisms, a tendency to decreased risk of EGJA was noted. In a subgroup analysis, a significantly decreased risk of EGJA in <64 years subgroup was identified. We found that *MTHFR* G_rs1801133_T_rs3753584_G_rs4845882_A_rs4846048_C_rs9651118_, G_rs1801133_C_rs3753584_A_rs4845882_A_rs4846048_T_rs9651118_ and G_rs1801133_T_rs3753584_A_rs4845882_G_rs4846048_T_rs9651118_ haplotypes significantly decreased the risk of EGJA (*P* = 0.002, *P* < 0.001 and *P* = 0.038, respectively). In conclusion, our study demonstrates that *MTHFR* rs1801133 G>A may be associated with the increased risk of EGJA. Meanwhile, *MTHFR* rs3753584 T>C, rs4845882 G>A and rs9651118 T>C polymorphisms and haplotypes may decrease the risk of EGJA in Eastern Chinese Han population. Further studies with large sample size and detailed gene-environmental data are needed to validate our conclusion.

## INTRODUCTION

The increasing incidence of esophagogastric junction adenocarcinoma (EGJA) was observed worldwide [1–3] and was considered to have different etiology and risk factor compared with distal gastric carcinoma (GC) [4]. EGJA remains poor prognosis [5] and is a common public health problem. The vital risk factors contributing to the development of EGJA are obesity, gastro-esophageal reflux disease, smoking, foods preserved by salting and low intake of fruits and vegetables *et al* [6, 7]. However, these observed risk factors could not interpret the overall susceptibility to EGJA. Recently, more and more epidemiologic studies suggested that individual’s genetic factor might influence the pathogenesis of EGJA.

Accumulating evidences indicate that folate insufficiency may increase the susceptibility of multiple malignancies [8, 9]. In humans, the majority of methyl groups may be presented by folic acid for endocellular methylation reactions and DNA de novo deoxynucleoside synthesis. During DNA synthesis, lack of folate can cause uracil misincorporation and then affect the stability of DNA [10]. In folate metabolism and DNA synthesis, methylenetetrahydrofolate reductase (MTHFR) is an important enzyme which catalyzes the revivification of 5,10-methylenetetrahydrofolate to 5-methyltetrahydrofolate. And 5-methyltetrahydrofolate is a main circulating and existing form of folate and is the methyl donor for DNA methylation and remethylation procedure of homocysteine to methionine. Based on the important role of participation in both DNA synthesis and methylation, any variant of *MTHFR* gene may involve in the carcinogenesis.

Human MTHFR is composed of 656 amino acids. *MTHFR* gene is located on the short arm of Chromosome 1. The human *MTHFR* gene is very polymorphic (http://www.ncbi.nlm.nih.gov/SNP) and a number of loci have been established, such as rs1537514, rs3753584, rs9651118, rs1537516, rs4845882, rs1801131, rs1801133, rs2066462, rs4846048 and rs3737967 polymorphisms, etc. Interestingly, many previous case-control studies demonstrated that *MTHFR* polymorphisms were correlated with the risk of multiple human malignancies [e.g., esophageal squamous cell carcinoma (ESCC) [11], gastric cardia adenocarcinoma (GCA) [12], cervicalcancer [13], breast cancer [14, 15] and childhood acute lymphoblastic leukemia [16] *et al*]. Thus, the single nucleotide polymorphisms (SNPs) in *MTHFR* genes on EGJA risk attracted our interest. Exploring the potential association of *MTHFR* SNPs with EGJA susceptibility may be conducive to the prevention and personalized diagnosis. In this study, we selected *MTHFR* tagging SNPs (rs1801133 G>A, rs3753584 T>C, rs4845882 G>A, rs4846048 A>G and rs9651118 T>C) and performed a case-control study to evaluate the effect of *MTHFR* genotypes for EGJA risk.

## RESULTS

### Baseline characteristics

A total of 1,063 sporadic patients with EGJA and 1,677 normal controls were recruited. Of the EJGA patients, 759 were male and 304 were female, with a mean age (± standard deviation) of 64.19 ±8.63 years. The normal controls comprised of 1,194 males and 483 females with a mean age of 63.91 ±10.22 years. The demographics (age and sex) was well matched (*P* = 0.165 and *P* = 0.909, respectively; Table 1). Of the smoking and alcohol consumption, a significant difference was observed between EGJA patients and controls (*P* < 0.001, Table 1). The frequency distribution of *MTHFR* genotypes was determined after genotyping the 2,740 study subjects. For *MTHFR* rs1801133 G>A, rs3753584 T>C, rs4845882 G>A, rs4846048 A>G and rs9651118 T>C polymorphisms, success rates of genotyping were 99.01%, 99.09%, 99.05%, 99.09% and 98.98%, respectively (Table 2). In controls, the distribution of *MTHFR* genotype frequencies accorded with Hardy–Weinberg equilibrium (HWE), except for *MTHFR* rs4846048 A>G polymorphism (Table 2).

**Table 1:** Distribution of selected demographic variables and risk factors in EGJA cases and controls

Variable	Overall Cases (n=1,063)	Overall Controls (n=1,677)	*P* ^a^
	n (%)	n (%)	
Age (years)	64.19 ±8.63	63.91 ±10.22	0.451
Age (years)			0.165
< 64	494 (46.47)	825 (49.19)	
≥64	569 (53.53)	852 (50.81)	
Sex			0.909
Male	759 (71.40)	1194 (71.20)	
Female	304 (28.60)	483 (28.80)	
Smoking status			**<0.001**
Never	773 (72.72)	1323 (78.89)	
Ever	290 (27.28)	354 (21.11)	
Alcohol use			**<0.001**
Never	908 (85.42)	1507 (89.86)	
Ever	155 (14.58)	170 (10.14)	

^a^ Two-sided *χ*^2^ test and Student *t* test.

**Table 2:** Primary information for *MTHFR* polymorphisms (rs1801133 G>A, rs3753584 T>C, rs4845882 G>A, rs4846048 A>G and rs9651118 T>C)

Genotyped SNPs	rs1801133 G>A	rs3753584 T>C	rs4845882 G>A	rs4846048 A>G	rs9651118 T>C
Chromosome	1	1	1	1	1
Function	Missense	NearGene-5	Intron	Intron	Intron
Chr Pos (Genome Build 36.3)	11778965	11787173	11765754	11768839	11784801
MAF^a^ for Chinese in database	0.439	0.093	0.198	0.105	0.382
MAF in our controls (n = 1,677)	0.359	0.108	0.209	0.096	0.378
*P* value for HWE^b^ test in our controls	0.679	0.691	0.972	0.014	0.270
Genotyping method	SNPscan	SNPscan	SNPscan	SNPscan	SNPscan
% Genotyping value	99.01%	99.09%	99.05%	99.09%	98.98%

^a^MAF: minor allele frequency.

^b^HWE: Hardy–Weinberg equilibrium.

### Association of *MTHFR* rs1801133 G>A, rs3753584 T>C, rs4845882 G>A, rs4846048 A>G and rs9651118 T>C polymorphisms with EGJA

The genotypes of *MTHFR* rs1801133 G>A, rs3753584 T>C, rs4845882 G>A, rs4846048 A>G and rs9651118 T>C polymorphisms are presented in Table 3. For *MTHFR* rs1801133 G>A polymorphism, the risk of developing EGJA was significant in three genetic models [AA *vs.* GG: crude odds ratio (OR) = 1.50, 95% confidence interval (CI): 1.19–1.90, *P* = 0.001; GA/AA *vs.* GG: crude OR = 1.27, 95% CI: 1.08–1.49, *P* = 0.004 and AA *vs.* GA/GG: crude OR = 1.45, 95% CI: 1.17–1.80, *P* = 0.001; Table 3]. Adjustment for age, sex, smoking and drinking, the similar results were also found (AA *vs.* GG: adjusted OR = 1.47, 95% CI: 1.16–1.86, *P* = 0.001; GA/AA *vs.* GG: adjusted OR = 1.25, 95% CI: 1.06–1.47, *P* = 0.007 amd AA *vs.* GA/GG: adjusted OR = 1.43, 95% CI: 1.15–1.77, *P* = 0.001; Table 3). For *MTHFR* rs4845882 G>A polymorphism, the decreased risk of EGJA was found in two genetic models (AA *vs.* GG: crude OR = 0.47, 95% CI: 0.29–0.75, *P* = 0.002 and AA *vs.* GA/GG: crude OR = 0.50, 95% CI: 0.31–0.80, *P* = 0.004; Table 3). Adjustment for age, sex, smoking and drinking, the results were not materially changed (AA *vs.* GG: adjusted OR = 0.47, 95% CI: 0.29–0.76, *P* = 0.002 and AA *vs.* GA/GG: adjusted OR = 0.50, 95% CI: 0.31–0.81, *P* = 0.005; Table 3). In addition, these associations were still significant after a Bonferroni correction for multiple comparisons.

**Table 3:** Logistic regression analyses of associations between *MTHFR* rs1801133 G>A, rs3753584 T>C, rs4845882 G>A, rs4846048 A>G and rs9651118 T>C polymorphisms and the risk of EGJA

Genotype	Cases (n=1,063)		Controls (n=1,677)		Crude OR (95%CI)	*P*	Adjusted OR ^a^ (95%CI)	*P*
	n	%	n	%				
*MTHFR* rs1801133 G>A								
GG	367	35.29	683	40.82	1.00		1.00	
GA	492	47.31	778	46.50	1.11 (0.94-1.32)	0.208	1.10 (0.93-1.30)	0.263
AA	181	17.40	212	12.67	**1.50 (1.19-1.90)**	**0.001**	**1.47 (1.16-1.86)**	**0.001**
GA + AA	673	64.71	990	59.18	**1.27 (1.08-1.49)**	**0.004**	**1.25 (1.06-1.47)**	**0.007**
GG+ GA	859	82.60	1,461	87.33	1.00		1.00	
AA	181	17.40	212	12.67	**1.45 (1.17-1.80)**	**0.001**	**1.43 (1.15-1.77)**	**0.001**
A allele	854	41.06	1,202	35.92				
*MTHFR* rs3753584 T>C								
TT	855	82.13	1,330	79.45	1.00		1.00	
CT	177	17.00	326	19.47	0.83 (0.67-1.01)	0.062	0.83 (0.68-1.01)	0.067
CC	9	0.86	18	1.08	0.76 (0.34-1.70)	0.504	0.73 (0.32-1.63)	0.440
CT+CC	186	17.87	344	20.55	0.84 (0.69-1.03)	0.087	0.84 (0.69-1.03)	0.091
TT+CT	1032	99.14	1,656	98.92	1.00		1.00	
CC	9	0.86	18	1.08	0.80 (0.36-1.79)	0.591	0.77 (0.34-1.72)	0.516
C allele	195	9.37	362	10.81				
*MTHFR* rs4845882 G>A								
GG	687	66.06	1,049	62.66	1.00		1.00	
GA	330	31.73	552	32.97	0.89 (0.75-1.05)	0.153	0.89 (0.75-1.05)	0.150
AA	23	2.21	73	4.36	**0.47 (0.29-0.75)**	**0.002**	**0.47 (0.29-0.76)**	**0.002**
GA+AA	353	33.94	625	37.34	0.86 (0.73-1.01)	0.074	0.86 (0.73-1.02)	0.075
GG+GA	1,017	97.79	1,601	95.64	1.00		1.00	
AA	23	2.21	73	4.36	**0.50 (0.31-0.80)**	**0.004**	**0.50 (0.31-0.81)**	**0.005**
A allele	376	18.08	698	20.85				
*MTHFR* rs4846048 A>G								
AA	860	82.61	1,378	82.32	1.00		1.00	
AG	171	16.43	272	16.25	0.98 (0.80-1.21)	0.883	0.99 (0.80-1.22)	0.921
GG	10	0.96	24	1.43	0.65 (0.31-1.37)	0.260	0.63 (0.30-1.34)	0.230
AG+GG	181	17.39	296	17.68	0.98 (0.80-1.20)	0.845	0.98 (0.80-1.21)	0.870
AA+AG	1,031	99.04	1,650	98.57	1.00		1.00	
GG	10	0.96	24	1.43	0.67 (0.32-1.40)	0.284	0.65 (0.31-1.36)	0.250
G allele	191	9.17	320	9.56				
*MTHFR* rs9651118 T>C								
TT	423	40.75	638	38.11	1.00		1.00	
TC	486	46.82	808	48.27	0.86 (0.73-1.02)	0.075	0.87 (0.74-1.03)	0.105
CC	129	12.43	228	13.62	0.81 (0.63-1.04)	0.094	0.83 (0.65-1.07)	0.150
TC+CC	615	59.25	1,036	61.89	0.90 (0.76-1.05)	0.171	0.91 (0.78-1.07)	0.260
TT+TC	909	87.57	1,446	86.38	1.00		1.00	
CC	129	12.43	228	13.62	0.90 (0.71-1.13)	0.372	0.92 (0.73-1.16)	0.489
C allele	744	35.84	1,264	37.75				

^a^ Adjusted for age, sex, smoking and drinking status; Bold values are statistically significant (*P* <0.05).

### Association of *MTHFR* rs1801133 G>A, rs3753584 T>C, rs4845882 G>A, rs4846048 A>G and rs9651118 T>C polymorphisms with EGJA in Different Stratification Groups

In the stratified analyses by sex, age, drinking and smoking, the genotype frequencies of *MTHFR* rs1801133 G>A polymorphism are listed in Table 4. After adjustment by logistic regression analysis, the association of *MTHFR* rs1801133 G>A variants with EGJA risk was evident in some subgroups [male group: AA *vs.* GG: adjusted OR = 1.66, 95% CI 1.26–2.20, *P* < 0.001, GA/AA *vs.* GG: adjusted OR = 1.27, 95% CI 1.05–1.53, *P* = 0.015 and AA *vs.* GA/GG: adjusted OR = 1.61, 95% CI 1.24–2.08, *P* < 0.001; <64 years subgroup: AA *vs.* GG: adjusted OR = 1.51, 95% CI 1.06–2.14, *P* = 0.022, GA/AA *vs.* GG: adjusted OR = 1.38, 95% CI 1.09–1.74, *P* = 0.007 and AA *vs.* GA/GG: adjusted OR = 1.39, 95% CI 1.00–1.92, *P* = 0.049; ≥64 years subgroup: AA *vs.* GG: adjusted OR = 1.42, 95% CI 1.03–1.95, *P* = 0.032 and AA *vs.* GA/GG: adjusted OR = 1.47, 95% CI 1.10–1.96, *P* = 0.010; never smoking group: AA *vs.* GG: adjusted OR = 1.43, 95% CI 1.08–1.87, *P* = 0.012, GA/AA *vs.* GG: adjusted OR = 1.32, 95% CI 1.10–1.59, *P* = 0.004 and AA *vs.* GA/GG: adjusted OR = 1.33, 95% CI 1.04–1.72, *P* = 0.026; ever smoking group: AA *vs.* GG: adjusted OR = 1.62, 95% CI 1.01–2.61, *P* = 0.046 and AA *vs.* GA/GG: adjusted OR = 1.79, 95% CI 1.15–2.76, *P* = 0.009 and never drinking group: AA *vs.* GG: adjusted OR = 1.54, 95% CI 1.20–1.98, *P* = 0.001, GA/AA *vs.* GG: adjusted OR = 1.35, 95% CI 1.13–1.60, *P* = 0.001 and AA *vs.* GA/GG: adjusted OR = 1.45, 95% CI 1.15–1.82, *P* = 0.002; Table 4)].

**Table 4:** Stratified analyses between *MTHFR* rs1801133 G>A polymorphism and EGJA risk by sex, age, smoking status and alcohol consumption

Variable	*MTHFR* rs1801133 G>A (case/control)^a^			Adjusted OR^b^ (95% CI); *P*				
	GG	GA	AA	GG	GA	AA	GA /AA	AA vs. (GA/GG)
Sex								
Male	260/485	350/563	135/142	1.00	1.10 (0.90-1.34); *P*: 0.360	**1.66 (1.26-2.20);** ***P*: < 0.001**	**1.27 (1.05-1.53);** ***P*: 0.015**	**1.61 (1.24-2.08);** ***P*: < 0.001**
Female	107/198	142/215	46/70	1.00	1.10 (0.80-1.50); *P*: 0.575	1.03 (0.66-1.60); *P*: 0.909	1.17 (0.86-1.59); *P*: 0.311	1.01 (0.67-1.53); *P*: 0.954
Age								
<64	173/362	233/363	76/98	1.00	1.24 (0.97-1.58); *P*: 0.089	**1.51 (1.06-2.14);** ***P*: 0.022**	**1.38 (1.09-1.74);** ***P*: 0.007**	**1.39 (1.00-1.92);** ***P*: 0.049**
≥64	194/321	259/415	105/114	1.00	0.97 (0.77-1.23); *P*: 0.822	**1.42 (1.03-1.95);** ***P*: 0.032**	1.12 (0.90-1.40); *P*: 0.312	**1.47 (1.10-1.96);** ***P*: 0.010**
Smoking status								
Never	263/549	368/603	123/168	1.00	1.19 (0.98-1.44); *P*: 0.085	**1.43 (1.08-1.87);** ***P*: 0.012**	**1.32 (1.10-1.59);** ***P*: 0.004**	**1.33 (1.04-1.72);** ***P*: 0.026**
Ever	104/134	124/175	58/44	1.00	0.86 (0.61-1.22); *P*: 0.396	**1.62 (1.01-2.61);** ***P*: 0.046**	1.04 (0.75-1.45); *P*: 0.805	**1.79 (1.15-2.76);** ***P*: 0.009**
Alcohol consumption								
Never	308/631	426/686	152/187	1.00	1.19 (0.99-1.42); *P*: 0.065	**1.54 (1.20-1.98);** ***P*: 0.001**	**1.35 (1.13-1.60);** ***P*: 0.001**	**1.45 (1.15-1.82);** ***P*: 0.002**
Ever	59/52	66/92	29/25	1.00	0.60 (0.36-1.00); *P*: 0.051	1.10 (0.56-2.19); *P*: 0.780	0.70 (0.43-1.13); *P*: 0.138	1.47 (0.80-2.73); *P*: 0.217

^a^ The genotyping was successful in 1063 (97.84%) EGJA cases, and 1677 (99.76%) controls for *MTHFR* rs1801133 G>A;

^b^ Adjusted for age, sex, smoking status and alcohol consumption (besides stratified factors accordingly) in a logistic regression model;

Table 5 summarizes the results of association between *MTHFR* rs3753584 T>C polymorphism and EGJA risk in the stratified analysis. We found that *MTHFR* rs3753584 T>C polymorphism was associated with the decreased risk of EGJA in <64 years subgroup [TC *vs.* TT: adjusted OR = 0.70, 95% CI 0.52–0.93, *P* = 0.016 and TC/CC *vs.* TT: adjusted OR = 0.73, 95% CI 0.55–0.97, *P* = 0.032 (Table 5)].

**Table 5:** Stratified analyses between *MTHFR* rs3753584 T>C polymorphism and EGJA risk by sex, age, smoking status and alcohol consumption

Variable	*MTHFR* rs3753584 T>C (case/control)^a^			Adjusted OR^b^ (95% CI); *P*				
	TT	TC	CC	TT	TC	CC	TC / CC	CC vs. (TC/TT)
Sex								
Male	613/950	126/226	7/15	1.00	0.85 (0.67-1.08); *P*: 0.177	0.69 (0.28-1.70); *P*: 0.415	0.85 (0.67-1.08); *P*: 0.184	0.72 (0.29-1.78); *P*: 0.471
Female	242/380	51/100	2/3	1.00	0.80 (0.55-1.17); *P*: 0.252	0.61 (0.08-4.43); *P*: 0.622	0.83 (0.57-1.20); *P*: 0.319	0.64 (0.09-4.72); *P*: 0.663
Age								
<64	398/640	79/177	5/7	1.00	**0.70 (0.52-0.93);** ***P*: 0.016**	0.99 (0.31-3.19); *P*: 0.987	**0.73 (0.55-0.97);** ***P*: 0.032**	1.08 (0.34-3.47); *P*: 0.899
≥64	457/691	98/149	4/11	1.00	0.98 (0.74-1.30); *P*: 0.881	0.53 (0.17-1.68); *P*: 0.282	0.97 (0.73-1.28); *P*: 0.813	0.54 (0.17-1.71); *P*: 0.296
Smoking status								
Never	619/1,058	131/249	4/14	1.00	0.88 (0.70-1.11); *P*: 0.283	0.47 (0.16-1.45); *P*: 0.189	0.88 (0.70-1.11); *P*: 0.288	0.49 (0.16-1.51); *P*: 0.216
Ever	236/272	46/77	5/4	1.00	0.74 (0.49-1.11); *P*: 0.144	1.22 (0.31-4.74); *P*: 0.776	0.77 (0.52-1.15); *P*: 0.202	1.29 (0.33-5.04); *P*: 0.711
Alcohol consumption								
Never	729/1,190	152/299	6/16	1.00	0.81 (0.65-1.00); *P*: 0.052	0.59 (0.23-1.52); *P*: 0.273	0.82 (0.66-1.01); *P*: 0.064	0.63 (0.24-1.61); *P*: 0.331
Ever	126/140	25/27	3/2	1.00	1.11 (0.60-2.06); *P*: 0.748	1.52 (0.24-9.63); *P*: 0.657	1.14 (0.63-2.07); *P*: 0.668	1.49 (0.24-9.44); *P*: 0.669

^a^ The genotyping was successful in 1063 (97.93%) EGJA cases, and 1677 (99.82%) controls for *MTHFR* rs3753584 T>C;

^b^ Adjusted for age, sex, smoking status and alcohol consumption (besides stratified factors accordingly) in a logistic regression model;

The results of association between *MTHFR* rs4845882 G>A polymorphism and EGJA risk in the stratified analyses are summarized in Table 6. We found that *MTHFR* rs4845882 G>A polymorphism decreased the risk of EGJA in several subgroups [male group: AA *vs.* GG: adjusted OR = 0.47, 95% CI 0.27–0.83, *P* = 0.009 and AA *vs.* GA/GG: adjusted OR = 0.50, 95% CI 0.29–0.87, *P* = 0.014; <64 years subgroup: AA *vs.* GG: adjusted OR = 0.41, 95% CI 0.20–0.84, *P* = 0.015 and AA *vs.* GA/GG: adjusted OR = 0.45, 95% CI 0.22–0.91, *P* = 0.027; never smoking group: AA *vs.* GG: adjusted OR = 0.37, 95% CI 0.21–0.67, *P* = 0.001 and AA *vs.* GA/GG: adjusted OR = 0.39, 95% CI 0.22–0.70, *P* = 0.002 and never drinking group: AA *vs.* GG: adjusted OR = 0.44, 95% CI 0.26–0.74, *P* = 0.002, GA/AA *vs.* GG: adjusted OR = 0.83, 95% CI 0.69–0.98, *P* = 0.032 and AA *vs.* GA/GG: adjusted OR = 0.48, 95% CI 0.29–0.80, *P* = 0.005 (Table 6)].

**Table 6:** Stratified analyses between *MTHFR* rs4845882 G>A polymorphism and EGJA risk by sex, age, smoking status and alcohol consumption

Variable	*MTHFR* rs4845882 G>A (case/control)^a^			Adjusted OR^b^ (95% CI); *P*				
	GG	GA	AA	GG	GA	AA	GA /AA	AA vs. (GA/GG)
Sex								
Male	492/746	237/391	17/54	1.00	0.89 (0.73-1.09); *P*: 0.268	**0.47 (0.27-0.83);** ***P*: 0.009**	0.86 (0.71-1.05); *P*: 0.113	**0.50 (0.29-0.87);** ***P*: 0.014**
Female	195/303	93/161	6/19	1.00	0.86 (0.63-1.18); *P*: 0.354	0.43 (0.16-1.12); *P*: 0.084	0.86 (0.63-1.17); *P*: 0.330	0.46 (0.18-1.21); *P*: 0.117
Age								
<64	320/507	152/279	10/37	1.00	0.83 (0.65-1.06); *P*: 0.132	**0.41 (0.20-0.84);** ***P*: 0.015**	0.81 (0.64-1.02); *P*: 0.077	**0.45 (0.22-0.91);** ***P*: 0.027**
≥64	367/542	178/273	13/36	1.00	0.94 (0.74-1.18); *P*: 0.566	0.53 (0.28-1.01); *P*: 0.052	0.91 (0.73-1.14); *P*: 0.424	0.55 (0.29-1.04); *P*: 0.068
Smoking status								
Never	496/832	243/427	14/62	1.00	0.92 (0.76-1.12); *P*: 0.416	**0.37 (0.21-0.67);** ***P*: 0.001**	0.89 (0.73-1.07); *P*: 0.207	**0.39 (0.22-0.70);** ***P*: 0.002**
Ever	191/217	87/125	9/11	1.00	0.81 (0.58-1.14); *P*: 0.220	0.96 (0.38-2.40); *P*: 0.927	0.83 (0.60-1.16); *P*: 0.269	1.04 (0.42-2.59); *P*: 0.939
Alcohol consumption								
Never	591/938	276/501	19/66	1.00	0.85 (0.71-1.01); *P*: 0.065	**0.44 (0.26-0.74);** ***P*: 0.002**	**0.83 (0.69-0.98);** ***P*: 0.032**	**0.48 (0.29-0.80);** ***P*: 0.005**
Ever	96/111	54/51	4/7	1.00	1.30 (0.80-2.12); *P*: 0.293	0.85 (0.23-3.19); *P*: 0.813	1.25 (0.78-2.02); *P*: 0.355	0.78 (0.21-2.87); *P*: 0.708

^a^ The genotyping was successful in 1063 (97.84%) EGJA cases, and 1677 (99.82%) controls for *MTHFR* rs4845882 G>A;

^b^ Adjusted for age, sex, smoking status and alcohol consumption (besides stratified factors accordingly) in a logistic regression model;

Table 7 lists *MTHFR* rs4846048 A>G genotype frequencies in the stratified analysis. We found no significant difference in genotype distribution of *MTHFR* rs4846048 A>G polymorphism among EGJA cases and non-cancer controls.

**Table 7:** Stratified analyses between *MTHFR* rs4846048 A>G polymorphism and EGJA risk by sex, age, smoking status and alcohol consumption

Variable	*MTHFR* rs4846048 A>G (case/control)^a^			Adjusted OR^b^ (95% CI); *P*				
	AA	AG	GG	AA	AG	GG	AG/GG	GG vs. (AG/AA)
Sex								
Male	615/984	124/189	7/18	1.00	1.04 (0.81-1.33); *P*: 0.772	0.58 (0.24-1.40); *P*: 0.227	1.02 (0.80-1.29); *P*: 0.904	0.58 (0.24-1.41); *P*: 0.233
Female	245/394	47/83	3/6	1.00	0.88 (0.59-1.30); *P*: 0.522	0.73 (0.18-2.96); *P*: 0.659	0.90 (0.61-1.32); *P*: 0.597	0.77 (0.19-3.11); *P*: 0.711
Age								
<64	398/677	78/134	6/12	1.00	0.99 (0.73-1.34); *P*: 0.927	0.74 (0.27-2.02); *P*: 0.562	0.99 (0.74-1.34); *P*: 0.955	0.76 (0.28-2.07); *P*: 0.590
≥64	462/701	93/138	4/12	1.00	1.00 (0.75-1.33); *P*: 0.981	0.48 (0.15-1.51); *P*: 0.210	0.97 (0.74-1.29); *P*: 0.855	0.49 (0.16-1.53); *P*: 0.221
Smoking status								
Never	624/1,081	125/220	5/20	1.00	0.96 (0.75-1.22); *P*: 0.725	0.43 (0.16-1.16); *P*: 0.095	0.94 (0.74-1.19); *P*: 0.611	0.45 (0.17-1.19); *P*: 0.107
Ever	236/297	46/52	5/4	1.00	1.13 (0.73-1.75); *P*: 0.582	1.49 (0.39-5.72); *P*: 0.563	1.17 (0.77-1.78); *P*: 0.473	1.47 (0.38-5.66); *P*: 0.572
Alcohol consumption								
Never	736/1,236	145/248	6/21	1.00	0.96 (0.77-1.20); *P*: 0.709	0.47 (0.19-1.16); *P*: 0.101	0.95 (0.76-1.18); *P*: 0.613	0.48 (0.19-1.19); *P*: 0.115
Ever	124/142	26/24	4/3	1.00	1.31 (0.70-2.46); *P*: 0.406	1.77 (0.36-8.68); *P*: 0.483	1.35 (0.74-2.46); *P*: 0.323	1.70 (0.35-8.32); *P*: 0.512

^a^ The genotyping was successful in 1063 (97.93%) EGJA cases, and 1677 (99.82%) controls for *MTHFR* rs4846048 A>G;

^b^ Adjusted for age, sex, smoking status and alcohol consumption (besides stratified factors accordingly) in a logistic regression model;

The results of relationship between *MTHFR* rs9651118 T>C polymorphism and EGJA risk in the stratified analyses are summarized in Table 8. We found that *MTHFR* rs9651118 T>C polymorphism was associated with the decreased risk of EGJA in <64 years subgroup [TC *vs.* TT: adjusted OR = 0.78, 95% CI 0.61–0.99, *P* = 0.040 (Table 8)].

**Table 8:** Stratified analyses between *MTHFR* rs9651118 T>C polymorphism and EGJA risk by sex, age, smoking status and alcohol consumption

Variable	*MTHFR* rs9651118 T>C (case/control)^a^			Adjusted OR^b^ (95% CI); *P*				
	TT	TC	CC	TT	TC	CC	TC / CC	CC vs. (TC/TT)
Sex								
Male	309/447	339/574	95/170	1.00	0.82 (0.68-1.00); *P*: 0.054	0.80 (0.60-1.07); *P*: 0.134	0.86 (0.71-1.04); *P*: 0.109	0.91 (0.69-1.19); *P*: 0.492
Female	114/191	147/234	34/58	1.00	1.03 (0.76-1.40); *P*: 0.857	0.99 (0.61-1.61); *P*: 0.970	1.11 (0.82-1.50); *P*: 0.501	1.01 (0.64-1.60); *P*: 0.956
Age								
<64	190/288	231/424	59/111	1.00	**0.78 (0.61-0.99);** ***P*: 0.040**	0.80 (0.55-1.15); *P*: 0.225	0.83 (0.66-1.05); *P*: 0.128	0.95 (0.68-1.34); *P*: 0.767
≥64	233/350	255/384	70/117	1.00	0.96 (0.76-1.21); *P*: 0.728	0.89 (0.63-1.25); *P*: 0.502	0.99 (0.80-1.23); *P*: 0.914	0.93 (0.68-1.28); *P*: 0.651
Smoking status								
Never	299/499	357/636	95/186	1.00	0.88 (0.73-1.06); *P*: 0.182	0.80 (0.60-1.06); *P*: 0.126	0.92 (0.77-1.11); *P*: 0.380	0.89 (0.68-1.16); *P*: 0.374
Ever	124/140	129/173	34/43	1.00	0.83 (0.59-1.16); *P*: 0.270	0.95 (0.56-1.59); *P*: 0.835	0.87 (0.63-1.19); *P*: 0.378	1.05 (0.64-1.72); *P*: 0.843
Alcohol consumption								
Never	357/564	415/728	113/213	1.00	0.86 (0.72-1.03); *P*: 0.092	0.80 (0.62-1.04); *P*: 0.096	0.90 (0.76-1.06); *P*: 0.214	0.89 (0.70-1.14); *P*: 0.369
Ever	66/74	71/80	16/15	1.00	0.93 (0.57-1.49); *P*: 0.750	1.12 (0.50-2.51); *P*: 0.783	0.97 (0.61-1.54); *P*: 0.907	1.17 (0.54-2.53); *P*: 0.686

^a^ The genotyping was successful in 1063 (97.65%) EGJA cases, and 1677 (99.82%) controls for *MTHFR* rs9651118 T>C;

^b^ Adjusted for age, sex, smoking status and alcohol consumption (besides stratified factors accordingly) in a logistic regression model;

### SNP haplotypes

We used a SHESIS software (http://analysis.bio-x.cn/myAnalysis.php) [17] to construct haplotypes of *MTHFR* gene (Table 9). Finally, five *MTHFR* haplotypes were identified. When *MTHFR* A_rs1801133_T_rs3753584_G_rs4845882_A_rs4846048_T_rs9651118_ haplotype was used as reference, we found that *MTHFR* G_rs1801133_T_rs3753584_G_rs4845882_A_rs4846048_C_rs9651118_, G_rs1801133_C_rs3753584_A_rs4845882_A_rs4846048_T_rs9651118_ and G_rs1801133_T_rs3753584_A_rs4845882_G_rs4846048_T_rs9651118_ haplotypes significantly decreased the risk of EGJA (*P* = 0.002, *P* < 0.001 and *P* = 0.038, respectively, Table 9).

**Table 9:** *MTHFR* haplotype frequencies (%) in patients and controls and risk of esophagogastric junction adenocarcinom

Haplotypes	Case (n=2,126)		Control (n=3,354)		Crude OR (95% CI)	*P*
	n	%	n	%		
A_rs1801133_T_rs3753584_G_rs4845882_A_rs4846048_T_rs9651118_	814	39.36	1169	34.92	1.00	
G_rs1801133_T_rs3753584_G_rs4845882_A_rs4846048_C_rs9651118_	708	34.24	1244	37.16	**0.82 (0.72-0.93)**	**0.002**
G_rs1801133_C_rs3753584_A_rs4845882_A_rs4846048_T_rs9651118_	171	8.27	335	10.01	**0.69 (0.56-0.85)**	**<0.001**
G_rs1801133_T_rs3753584_A_rs4845882_G_rs4846048_T_rs9651118_	173	8.37	309	9.23	**0.80 (0.65-0.99)**	**0.038**
G_rs1801133_T_rs3753584_G_rs4845882_A_rs4846048_T_rs9651118_	130	6.29	206	6.15	0.91 (0.72-1.15)	0.416
Others	72	3.48	85	2.54	1.22 (0.88-1.69)	0.239

## DISCUSSION

Incidence of EGJA has increased over the past two decades [18, 19]. Many studies demonstrated that the morbidity of EGJA was increased in Asian countries, such as China, Korea and Japan [19–21]. However, the etiology of EGJA remains unknown. In this study, we explored the association between *MTHFR* rs1801133 G>A, rs3753584 T>C, rs4845882 G>A, rs4846048 A>G and rs9651118 T>C polymorphisms and EGJA risk in Eastern Chinese Han population. We found that *MTHFR* rs1801133 G>A might be associated with the increased risk of EGJA. Meanwhile, *MTHFR* rs3753584 T>C, rs4845882 G>A and rs9651118 T>C polymorphisms decreased the risk of EGJA.

*MTHFR* gene lies in 1p36.3 and contains 11 exons with a length of about 1980 bp. In exon 4, a G to A variant at nucleotide 677 locus (rs1801133 G>A) directly leads to valine substitution for alanine, which is relevant to a reduction of MTHFR activity [22]. The individuals who carry heterozygous genetype (GA genetype) of *MTHFR* rs1801133 G>A polymorphism have 70% of normal enzyme activity, however, those who carry homozygous genotype (AA genetype) have only 30% of normal enzyme activity [23]. A case-control study reported that rs1801133 AA genotype was associated an increased risk of GCA [24]. Another case-control study also found that *MTHFR* rs1801133 AA and GA genotypes were associated the increased risk of GCA [25]. These results were in accordance with our conclusions. In the future, more replicated study should be conducted to verify these primary findings.

*MTHFR* rs3753584 T>C is situated in the intron region of *MTHFR* gene. There were only a few studies focusing on the association between *MTHFR* rs3753584 T>C and cancer risk. A previous study found that there was an increased lung cancer risk in carriers of *MTHFR* rs3753584 CC genotype compared with carriers of rs3753584 TT genotype [26]. However, no association was found between ESCC risk and *MTHFR* rs3753584 T>C polymorphism [11]. In addition, Wang *et al.* also reported that *MTHFR* rs3753584 T>C was not associated with GCA risk [12]. The present study concluded that rs3753584 TC and TC/CC genotypes were related to a decreased EGJA risk in <64 years subgroup. These apparent discrepancy findings may be due to the insufficient sample size. In the future, more studies with large sample size and detailed environmental factors are indispensable to explore the relationship between *MTHFR* rs3753584 T>C and the risk of different cancers.

*MTHFR* rs4845882 G>A polymorphism lies in a intron region and is almost complete linkage disequilibrium (LD) with *MTHFR* rs1801131 A>C locus. Shen *et al.* found there was no significant relationship between *MTHFR* rs4845882 G>A polymorphism and gastric cancer risk [27]. Additionally, the association between *MTHFR* rs4845882 G>A and GCA risk was not concluded in a recent sutdy [12]. However, our study saw a decreased EGJA risk in the individuals carrying *MTHFR* rs4845882 AA genotype in male and <64 years subgroups. *MTHFR* rs9651118 T>C is situated in intron 2 and possesses low LD with rs1801133 G>A (*r*^2^ < 0.30). Fuctional annotation by HapReg demonstrated that *MTHFR* rs9651118 T>C coincides with *MTHFR* enhancers or promoters, which may correspond to the regions of open chromatin [28]. Several studies implicated that *MTHFR* rs9651118 C allele was associated with a reduced risk of lung cancer and prostate cancer [28, 29]. In addition, *MTHFR* rs9651118 C allele was associated with a decreased risk of breast cancer [30]. Our results suggested that *MTHFR* rs9651118 TC genotype may reduce EGJA susceptibility in <64 years subgroup, which were very similar to the findings of previous studies. In the future, these potential should be confirmed by functional studies.

In this case-control study, we constructed five *MTHFR* haplotypes to assess the potential inherited patterns of haplotype. We found that *MTHFR* G_rs1801133_T_rs3753584_G_rs4845882_A_rs4846048_C_rs9651118_, G_rs1801133_C_rs3753584_A_rs4845882_A_rs4846048_T_rs9651118_ and G_rs1801133_T_rs3753584_A_rs4845882_G_rs4846048_T_rs9651118_ haplotypes significantly decreased the risk of EGJA. To the best of our knowledge, we first explore the relationship of haplotypes in *MTHFR* rs1801133 G>A, rs3753584 T>C, rs4845882 G>A, rs4846048 A>G and rs9651118 T>C polymorphisms with EGJA susceptibility. We also found that *MTHFR* rs1801133 G and rs4845882 A alleles might be protective factors for haplotype to EGJA.

However, several limitations in our study should be presented. First, for the controls were recruited from the local hospitals, the selection bias of the study population should not be ignored. Second, the data of plasma folate level were not available, which may affect the association between *MTHFR* SNPs and EGJA susceptibility. Thirdly, for lack of cancer stage, disease progression and overall survival data, we did not consider the influence of *MTHFR* SNPs on progress and prognosis of EGJA. Last but not least, other environmental and genetic factors were not considered. Further studies are necessary to explore the effect of interactions between environment and gene factors on EGJA risk.

In conclusion, our study demonstrates that *MTHFR* rs1801133 G>A may be associated with the increased risk of EGJA. Meanwhile, *MTHFR* rs3753584 T>C, rs4845882 G>A and rs9651118 T>C polymorphisms decrease the risk of EGJA in Eastern Chinese Han population. The further case-control studies are needed to confirm our findings.

## MATERIALS AND METHODS

### Subjects

Study conducted at the Affiliated Union Hospital of Fujian Medical University, Fujian Medical University Cancer Hospital and the Affiliated People’s Hospital of Jiangsu University was approved by the Ethics Committee of Fujian Medical University (Fuzhou, China) and Jiangsu University (Zhenjiang, China). Subjects were enrolled from three hospitals in Eastern China. Our study involved 2,740 study participants, comprising 1,063 histopathologically confirmed sporadic EGJA patients and 1,677 healthy normal controls. Among them, 280 EGJA patients and 840 controls were enrolled from Fujian Medical University Union Hospital and Cancer Hospital of Fujian Medical University from January 2014 to May 2016. In addition, 783 EGJA patients and 837 controls were enrolled from the Affiliated People’s Hospital of Jiangsu University between January 2008 and November 2016. All EJGA patients were Siewert type II. The control group involved normal individuals who visited these hospitals for health check. The healthy normal controls were unrelated to the EGJA patients and were cancer-free individuals. Data of demographic details and risk factors was obtained using a structured questionnaire. The definition of ‘ever smokers’ were subjects who smoked at least one cigarette per day over 1 year [11], and ‘ever drinkers’ were subjects who drank no less than three times a week for more than 6 months [11]. The corresponding data are listed in Table 1. The Ethical Committee of Fujian Medical University and Jiangsu University approved the study protocols (No. SQ2015-006-01 and No. 20150083, respectively).

### Selection of SNPs

The *MTHFR* tagging SNPs (upstream and downstream of *MTHFR* gene extending 5 Kb, respectively) were selected from the database of CHB population using the HapMap Project (http://hapmap.ncbi.nlm.nih.gov/index.html.en) and Haploview 4.2 software. The major criterion were: (a) MAF ≥ 0.05 and call rate ≥ 95 %, (b) a HWE *P* ≥ 0.05, (c) a pairwise linkage disequilibrium (LD) *r*^*2*^ threshold of 0.8 between polymorphisms (*r*^2^ > 0.8) [11, 31, 32]. Finally, five *MTHFR* tagging SNPs (rs1801133 G>A, rs3753584 T>C, rs4845882 G>A, rs4846048 A>G and rs9651118 T>C) were eligible and included in this case-control study to evaluate the effect of *MTHFR* polymorphisms with EGJA risk. The primary information of *MTHFR* tagging SNPs is presented in Table 2.

### DNA extraction and genotyping

Each participant donated 2ml blood sample which was stored in an EDTA-anticoagulated tube. We use the Promega Genomic DNA Purification Kit (Promega, Madison, USA) to extract the genomic DNA. SNPscan™ genotyping assay (Genesky Biotechologies Inc., Shanghai, China) [33, 34] was harnessed to determine the genotyping of *MTHFR* rs1801133 G>A, rs3753584 T>C, rs4845882 G>A, rs4846048 A>G and rs9651118 T>C polymorphisms. Briefly, 150ng DNA sample was denatured at 98°C for 5min. The ligation reaction was done in an ABI 2720 thermal cycler. We used a 48-plex fluorescence PCR reaction for each ligation product amplification. In an ABI 3730XL sequencer, PCR products were analyzed by capillary electrophoresis. The obtained raw data were conducted by GeneMapper 4.1 software (Applied Biosystems, USA). One hundred and ten DNA samples (4%) were randomly selected to reanalyze the genotypes by different laboratory technicians and the reproducibility was 100%.

### Statistical analysis

Age of EGJA patients and controls was expressed as mean ± standard deviation. And a Student’s t-test was harnessed to assess the difference for age. The Chi-square test (*χ*^2^) was used to compare age, sex, smoking, drinking and the genotypes distribution of *MTHFR* SNPs in patients and controls. We used multivariate logistic regression analysis to assess the risk of *MTHFR* rs1801133 G>A, rs3753584 T>C, rs4845882 G>A, rs4846048 A>G and rs9651118 T>C polymorphisms and considered the confounders such as sex, age, smoking and drinking status. The crude/adjusted ORs and 95% CIs were calculated using the SAS software (Version 9.4; SAS Institute Inc., Cary, NC, USA). A *P* < 0.05 (two sided) was considered as statistical significance. In this study, multiple comparisons were conducted by Bonferroni correction [35]. We used a SHESIS software (http://analysis.bio-x.cn/myAnalysis.php) [17] to construct *MTHFR* haplotypes.
